# Precise repair of *mPing* excision sites is facilitated by target site duplication derived microhomology

**DOI:** 10.1186/s13100-015-0046-4

**Published:** 2015-09-07

**Authors:** David M. Gilbert, M. Catherine Bridges, Ashley E. Strother, Courtney E. Burckhalter, James M. Burnette, C. Nathan Hancock

**Affiliations:** Department of Biology and Geology, University of South Carolina Aiken, 471 University Parkway, Aiken, SC 29801 USA; Present Address: Department of Pathology and Laboratory Medicine, Medical University of South Carolina, Charleston, SC 29425 USA; Present Address: College of Natural and Agricultural Sciences, University of California Riverside, Riverside, CA 92521 USA

**Keywords:** *mPing*, Excision site repair, Target site duplication

## Abstract

**Background:**

A key difference between the *Tourist* and *Stowaway* families of miniature inverted repeat transposable elements (MITEs) is the manner in which their excision alters the genome. Upon excision, *Stowaway*-like MITEs and the associated *Mariner* elements usually leave behind a small duplication and short sequences from the end of the element. These small insertions or deletions known as “footprints” can potentially disrupt coding or regulatory sequences. In contrast, *Tourist*-like MITEs and the associated *PIF/Pong*/*Harbinger* elements generally excise precisely, returning the genome to its original state. The purpose of this study was to determine the mechanisms underlying these excision differences, including the role of the host DNA repair mechanisms.

**Results:**

The transposition of the *Tourist*-like element*, mPing*, and the *Stowaway*-like element, *14T32*, were evaluated using yeast transposition assays. Assays performed in yeast strains lacking non-homologous end joining (NHEJ) enzymes indicated that the excision sites of both elements were primarily repaired by NHEJ. Altering the target site duplication (TSD) sequences that flank these elements reduced the transposition frequency. Using yeast strains with the ability to repair the excision site by homologous repair showed that some TSD changes disrupt excision of the element. Changing the ends of *mPing* to produce non-matching TSDs drastically reduced repair of the excision site and resulted in increased generation of footprints.

**Conclusions:**

Together these results indicate that the difference in *Tourist* and *Stowaway* excision sites results from transposition mechanism characteristics. The TSDs of both elements play a role in element excision, but only the *mPing* TSDs actively participate in excision site repair. Our data suggests that *Tourist*-like elements excise with staggered cleavage of the TSDs, which provides microhomology that facilitates precise repair. This slight modification in the transposition mechanism results in more efficient repair of the double stranded break, and thus, may be less harmful to host genomes by disrupting fewer genes.

**Electronic supplementary material:**

The online version of this article (doi:10.1186/s13100-015-0046-4) contains supplementary material, which is available to authorized users.

## Background

Type II DNA transposable elements (TE) are present in most, if not all, eukaryotic genomes, but are especially abundant in plants where they play a role in genome evolution [1]. Plant DNA TEs have been classified into superfamilies including *hAT*, *MuDR*/*MU*, *CACTA*, *Mariner*, and *Harbinger*/*Pong* [2]. Each of these superfamilies is composed of autonomous elements that encode the proteins required for mobilization and non-autonomous elements that can only be mobilized *in trans* [3, 4]. Of special interest are the small (<500 bp) non-autonomous miniature inverted repeat TEs (MITEs). These are the most abundant TEs in the genome, often reaching thousands of copies, due to their ability for rapid proliferation [5–7]. The two best characterized MITE families, *Stowaway* and *Tourist*, have unique characteristics stemming from differences in their transposition mechanisms. *Stowaway-*like MITEs are mobilized by transposase proteins encoded by autonomous *Mariner-*like elements, produce a 2 bp target site duplication (TSD) upon insertion, and commonly leave small insertions or deletions (footprints) at their excision site [8]. *Tourist*-like MITEs are mobilized by transposase proteins encoded by the autonomous *PIF/Pong*-like elements, produce a 3 bp TSD, and generally excise precisely leaving no footprints at their excision site [9].

DNA TEs and their associated MITEs are mobilized by a “cut and paste” mechanism in which transposase proteins bind to the terminal inverted repeats (TIRs), effectively positioning the catalytic domain for the DNA cleavage that is required for both excision and insertion [10]. Staggered cleavage of the genomic DNA at the insertion site results in either a 5′ or 3′ overhang, both of which create small TSDs that flank the inserted elements. Based on the fact that *Mariner*-like and *Stowaway*-like elements have 2 bp TSDs, the transposase proteins likely produce a 2 bp overhang upon cleavage of the DNA [8]. The *PIF/Pong*-like and *Tourist*-like elements have 3 bp TSDs, indicating cleavage by their encoded transposases produce a 3 bp overhang [11]. Analysis of the excision sites of the elements can elucidate differences in the catalytic mechanism of their specific transposases. For example, the excision sites of the *Ac* and *Ds* elements in both plants and yeast demonstrate that their footprints are palindromic sequences from the flanking DNA, as opposed to pieces of the TE itself [12–14]. This suggests that this transposase cleaves at the end of the element, causing hairpin formation at the ends of the double stranded break. In contrast, the excision sites of *Mariner*/*Stowaway*-like elements contain footprints that often include some of the sequences of the element in addition to retaining the TSDs [15]. This indicates that that the *Mariner*-like transposase cleaves with a staggered cut at the end of the TIR for excision, leaving behind the TSD and a short region of single stranded TIR [15].

Excision of these DNA TEs produces double stranded breaks that are repaired by the host DNA repair mechanisms. This can be accomplished using a complementary template for homologous recombination (HR) or by the non-homologous end joining (NHEJ) pathway [16]. In plants, excision site analysis indicates that many of the repaired sites include insertions or deletions consistent with NHEJ [17–19]. In addition, yeast transposition experiments with the *Ac* element superfamily showed that repair of the double stranded break after excision required NHEJ proteins [12]. This study also showed that microhomology (<6 bp) exposed by end processing between the two strands flanking the element is often used to facilitate repair [12]. Differences in the proteins required for the repair of these ends may hint at the nature of the DNA breaks produced by the different transposases.

The focus of this study was to further characterize the transposition mechanism of the best studied *Tourist*-like MITE, *mPing*. In contrast to the previously mentioned elements, *mPing* excision sites are repaired precisely (leaving no element or TSD sequences). Based on these unique excision sites, we hypothesize that the *mPing* transposase proteins may cut at the TSD sequences adjacent to the element instead of within the element as seen for *Mariner*-like elements. Because the TSD sequences are identical, staggered cleavage at this location produces compatible sticky ends, providing microhomology for NHEJ that would easily restore the genome back to its original state before insertion of the element. Based on this hypothesis, we predict that alteration of *mPing’s* TSDs would alter the microhomology and reduce the effectiveness of NHEJ repair. Using a previously developed yeast transposition assay [20, 21], we tested the result of changing the TSDs for a *Tourist*-like MITE (*mPing*) and *Stowaway*-like MITE (*OsMar 14T32* or the hyperactive *OsMar 14T32-T7*). By performing these assays in yeast strains with a defective NHEJ DNA repair pathway, we were able to distinguish between impaired element excision and DNA repair.

## Results and discussion

### NHEJ is used for excision site repair

**Fig. 1:**
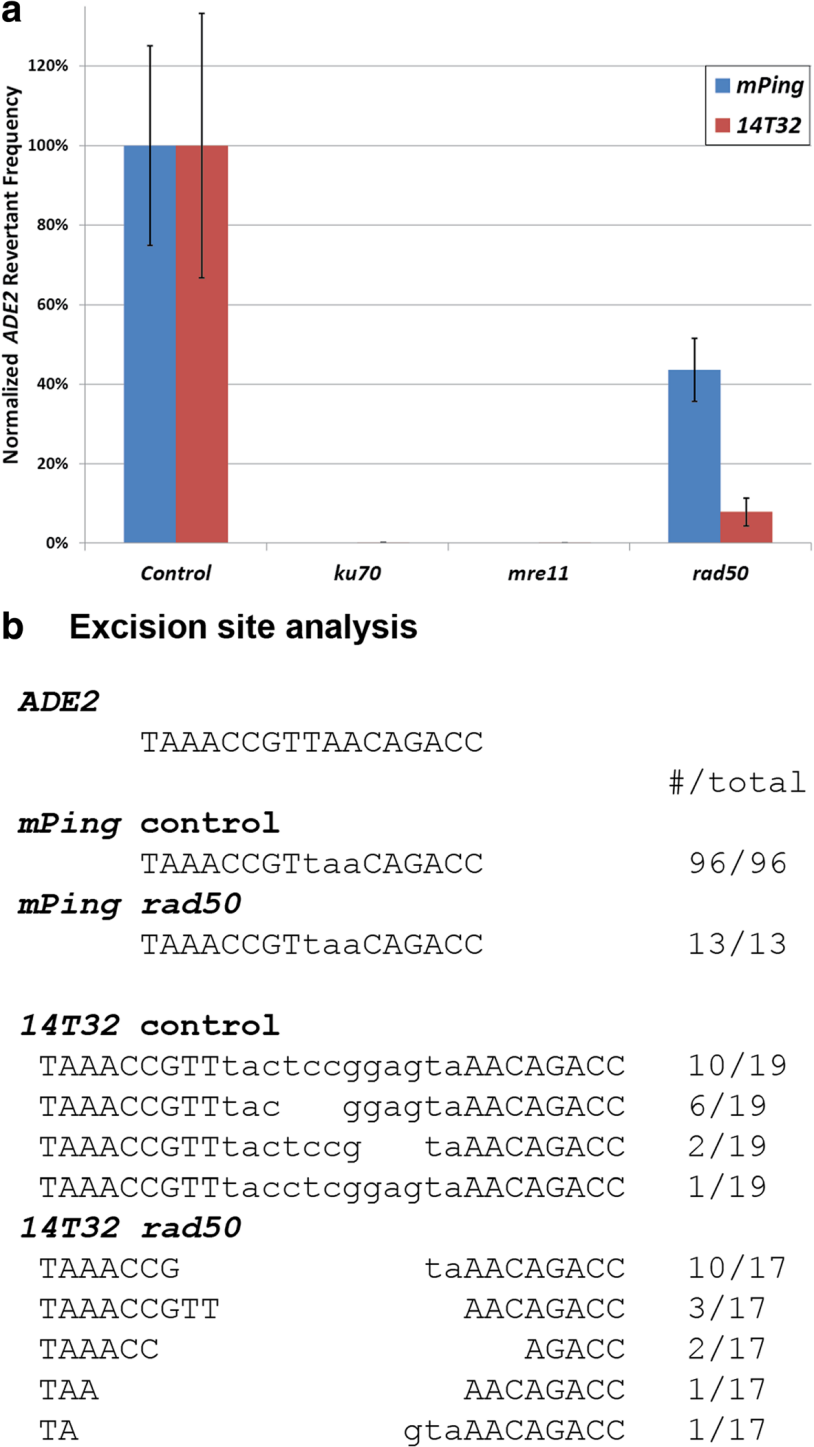
Transposition assays in NHEJ deficient yeast. Normalized *ADE2* revertant frequency for the *mPing* (*blue*) and *14T32* (*red*) elements in control (JIM17) and NHEJ mutant yeast strains (**a**). Error bars indicate the standard error for 6 replicates. Repaired excision sites from control and *rad50* yeast strains (**b**). Lowercase letters indicate the bases derived from the TSD (*mPing*) or TIRs and TSDs (*14T32*)

The yeast transposition assay used for these experiments measures the rate at which the *ADE2* gene is repaired in-frame following excision of the TE (Additional file 1) [14, 15, 20, 21]. Traditionally, these assays have been performed in haploid yeast lacking an *ADE2* homologous template for HR repair of the excision site. Under these conditions the excision site should be repaired only by NHEJ. Performing transposition assays with *mPing* and *14T32* in haploid yeast strains lacking the NHEJ pathway proteins *KU70*, *MRE11*, or *RAD50* showed that these proteins are required for efficient repair of the excision sites of both elements (Fig. 1a). Almost no *ADE2* revertant colonies were obtained in the *ku70* strain, as *KU70* is a highly conserved protein involved in the initial binding of the double stranded breaks [22]. For both elements, the *rad50* strain showed a higher DNA repair rate than the *mre11* strain. This is consistent with a previous study indicating that *MRE11* function is more important for repair than *RAD50* even though these two proteins function together in the MRX complex to process double stranded breaks before ligation [22, 23]. These results also indicated *RAD50* plays a more important role in excision site repair for the *14T32* element than the *mPing* element [92 % vs. 56 % decrease in repair efficiency (Fig. 1a)]. However, some of this change could be due to a difference in the amount of repair products that result in reading frame disruption. Analysis of excision sites produced in the *rad50* background showed that the *mPing* excision sites were still repaired precisely, while the *14T32* excision sites had more bases deleted (less precise repair) compared to the control (Fig. 1b). This difference in repair efficiency and quality observed for the two elements in the *rad50* strain provides evidence that there are important differences in the nature of the double strand breaks produced by these two elements.

**Fig. 2:**
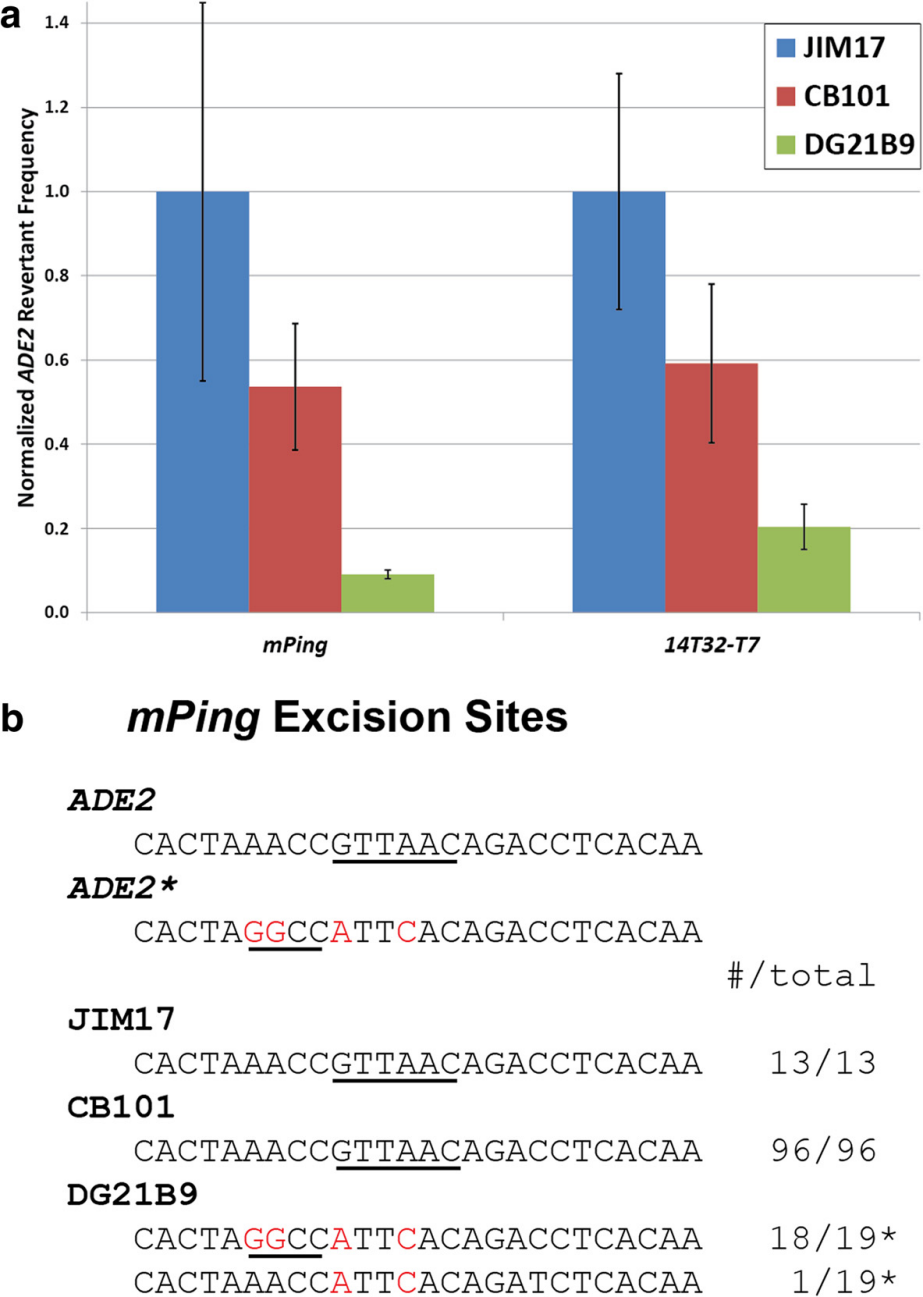
Transposition assays in yeast with altered DNA repair potentials. *ADE2* revertant frequencies for the *mPing* and *14T32-T7* elements in yeast strains with different DNA repair mechanisms available for excision site repair (**a**). JIM17 repairs by NHEJ, CB101 is capable of both HR and NHEJ, and DG21B9 can only repair by HR. Frequencies were normalized to the activity of each transposable element in JIM17. Error bars represent standard error. Sequences identified at the *mPing* (5′ TAA/3′ TAA TSDs) excision sites by restriction site analysis and sequencing (**b**). Underlined sequences indicate the *Hpa*I and *Hae*III sites used for analysis. Red bases are unique to the ADE2* template. *indicates the excision site was repaired by HR using the ADE2* template

Performing yeast transposition assays in a yeast strain that provides a partial *ADE2* template is an effective strategy to evaluate whether HR can be used for excision site repair. This approach has been used to study the *Ac* element (*hAT* superfamily, also creates footprints upon excision) where it was reported that when a template is available, about half of the excision sites are repaired by HR [12]. In this study, we employed similar methodology to determine if *mPing* and the hyperactive *OsMar 14T32-T7* excision sites are repaired by HR; we used the CB101 yeast strain that contains a partial *ADE2* template called ADE2*. This experiment showed that while no significant difference in the rate of *ADE2* revertant colonies is observed with or without the ADE2* template, CB101 seems to show slightly lower average *ADE2* revertants (Fig. 2). This may be due to competition between the two repair pathways or some unknown genetic change present in CB101. This slight difference did not affect our experiments because we were able to normalize within strains. To determine if HR was occurring in this strain, we analyzed 96 *mPing* excision sites and fifteen *14T32-T7* excision sites by PCR and digestion with *Hae*III (present in ADE2* but not in the original *ADE2*). Under these conditions, none of the excision sites in either element contained the *Hae*III site, and thus, were not repaired by HR at detectable levels (Fig. 2b). This indicates that even when a homologous template is present, the predominant repair pathway for these excision sites is NHEJ.

In order to allow separate analysis of element excision and repair, we developed a yeast strain (DG21B9) that was capable of performing HR at the excision site (contained the ADE2* template), but also had an impaired NHEJ pathway (*ku70*). In this strain, the number of *ADE2* revertant colonies was drastically reduced for both *mPing* and *14T32-T7* (Fig. 2), but was still higher than observed in the absence of a homologous template (Fig. 1). This drop in activity in this NHEJ deficient strain was consistent with the finding that NHEJ is the dominant pathway for repair of the excision sites. This together with the results for CB101 suggest that HR repair of these breaks functions as a backup to NHEJ and only occurs at about 10–20 % of the rate of NHEJ repair. Analysis of the DG219B *ADE2* revertant *mPing* excision sites by digestion and sequencing showed that 100 % were repaired by HR (Fig. 2). Most of these excision sites (18/19) contained the ADE2* specific *Hae*III site and the remaining site showed that ADE2* was used in such a way as to only remove the *Hpa*I site and not add the *Hae*III site (Fig. 2).

The ability to perform transposition assays in this NHEJ deficient strain (DG21B9) makes it possible to exclude the effects that the quality (i.e. blunt, staggered cut, presence or absence of microhomology) of the DNA break has on repair efficiency. This is because HR is less dependent on the immediate sequence at the end of the double stranded break, instead using sequences farther away from the cleavage site. Thus, this strain provides a method to differentiate whether a mutation affects the rate of NHEJ repair or the rate of excision.

### TSD alteration disrupts element excision

**Fig. 3:**
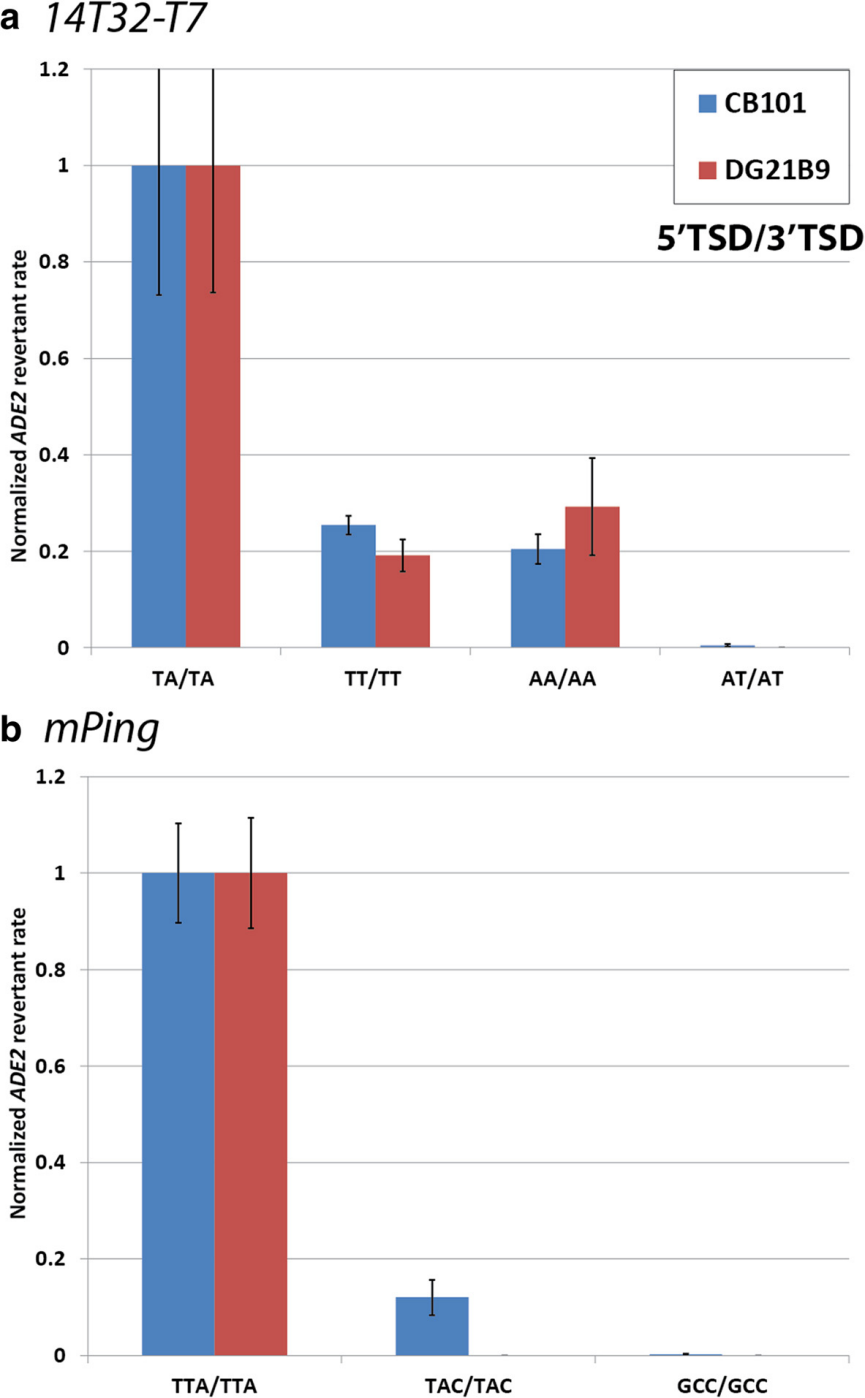
Transposition assays with altered but matching TSDs. *ADE2* revertant rates for *14T32-T7* (**a**) and *mPing* (**b**) elements with altered but matching TSDs. Blue bars indicate the rate in CB101 (capable of both NHEJ and HR), while red bars indicate the rate in DG21B9 (only capable of HR). Values were normalized to the control TSDs (TA/TA for *14T32-T7* and TAA/TAA for *mPing*) for each yeast strain separately. Error bars represent standard error

Previous studies have shown that *Mariner*-like elements require the TSD (TA on both ends) for transposition in vitro [24]. In this study, we confirmed the importance of the conserved TSD for the 1*4T32-T7* element by changing the TSDs and performing yeast transposition assays. In CB101, changing both bases of the TSDs from TA/TA (5′/3′) to AT/AT almost completely inhibited transposition, while changing just one base (TT/TT or AA/AA) allowed transposition, but at highly reduced rates (Fig. 3a). This experiment was also performed in DG21B9 (HR competent, NHEJ deficient) to confirm that this decrease in activity was due to inhibited excision and not inhibited excision site repair. Figure 3a shows that in DG21B9 alteration of the *14T32-T7* TSDs produced a comparable decrease in activity to the one observed in CB101 yeast. Thus, the drop in activity upon changing the TSDs is likely due to a decrease in excision, and not due to changes in the efficiency of NHEJ. Other researchers have shown that the *Mariner*-like transposase proteins bind to the TIRs and not the TSDs [25, 26]. Therefore, the TSDs do not likely play a role in binding, but instead play a role in the catalytic mechanism that cleaves the element from the genome.

To determine what role the TSDs play in *mPing* transposition, we performed yeast assays with *mPing* elements with altered TSDs. These experiments indicate that alteration of *mPing*’s TSDs also inhibits its transposition (Fig. 3b, Additional file 2a). Based on insertion site analysis, it was already known that T or A was acceptable at the middle position of the TSD [7]. Changing the middle base to C or G (i.e. from TAA/TAA (5′/3′) to TCA/TCA) had a small effect with TGA/TGA TSDs producing more colonies than TCA/TCA TSDs (Additional file 2a). Changing the first base (i.e. GAA/GAA) or third base (i.e. TAC/TAC) caused a more severe drop in the number of *ADE2* revertants. Changing all three bases completely disrupted the transposition of the element (Fig. 3b, Additional file 3a). To determine if this decrease in *ADE2* revertants was caused by a drop in excision or from a decreased rate of repair, a subset of these altered elements were tested in the DG21B9 strain (HR only). If altering the TSDs to this extent only affects repair of the excision site and not excision itself, all of these altered TSDs would have the same *ADE2* revertant rate as the control in DG21B9. However, almost no *ADE2* revertant colonies were detected in the TAC/TAC or GCC/GCC TSD (5′/3′) combinations (Fig. 3b), indicating that these base changes inhibit the ability of the transposase proteins to catalyze excision. It is not clear if this is due to altered enzyme binding or if these bases are directly involved in the catalytic mechanism.

In addition to reducing the number of *ADE2* revertant colonies by decreasing excision, sequencing the excision sites indicated that altering the TSDs can result in imprecise repair (Additional file 3b). The production of footprints was especially pronounced for the TAC/TAC TSDs, with 10 of 16 excision sites having indels. The inefficient excision of these altered elements may have resulted in strand cleavage in a non-standard position, creating double stranded breaks that were not as easily repaired.

### *mPing* excision site repair is facilitated by TSD homology

**Fig. 4:**
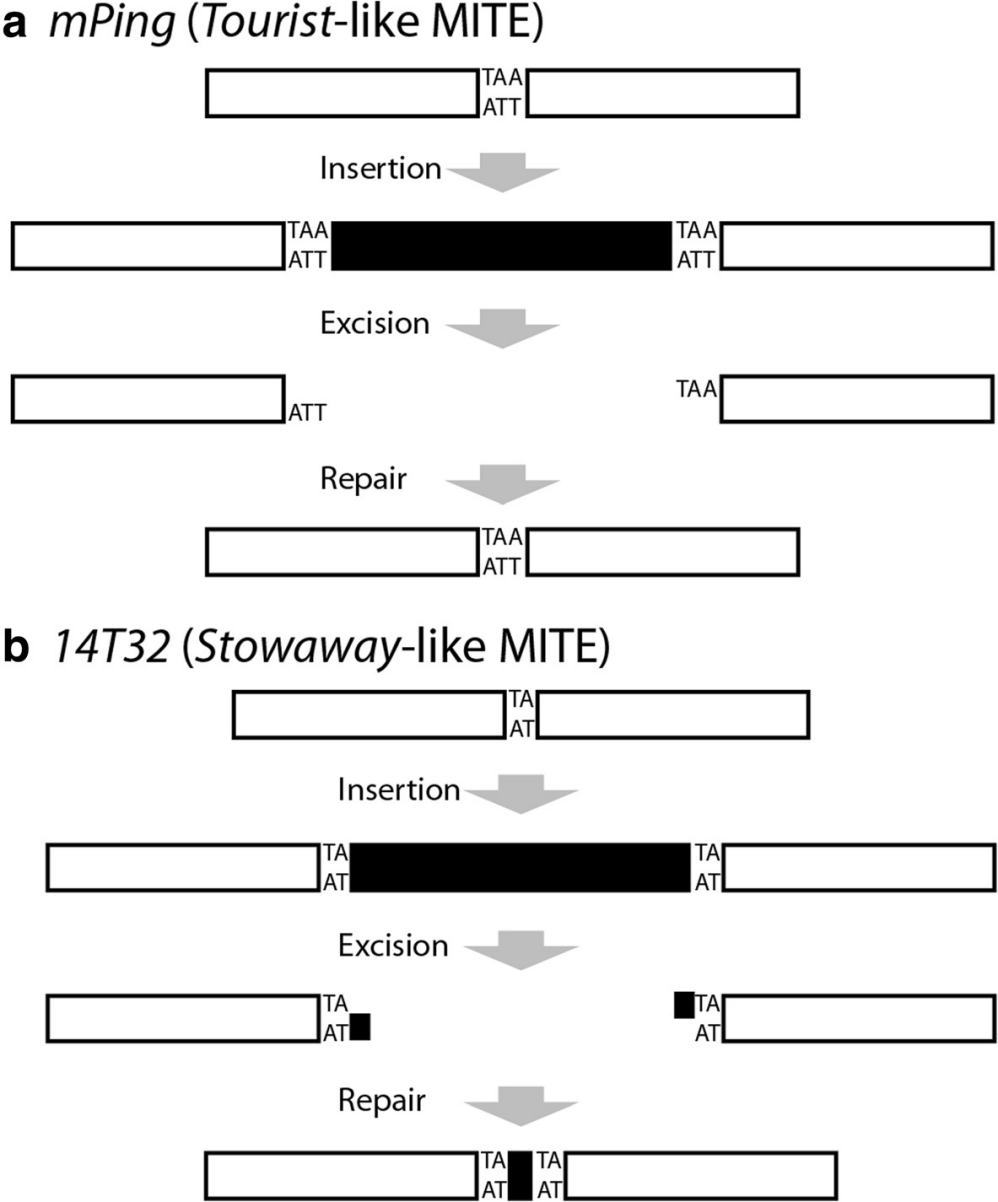
Model of *Tourist*-like and *Stowaway*-like MITE transposition. *mPing* (**a**) and *14T32-T7* (**b**) elements are represented by black boxes, with the TSDs (3 bp and 2 bp respectively) created upon insertion shown as letters. Excision of the *mPing* element produces TSD derived 5′ overhangs that result in precise repair, while *14T32* excision leaves element derived overhangs that results in footprint production

Based on these initial experiments, we hypothesized that a difference in the double stranded breaks created by the *mPing* and *14T32* elements results in their excision site differences*.* Analysis of repaired excision sites shows that *Mariner*-like transposase proteins produce staggered DNA cleavage within the element, leaving behind some of the TIR sequences (Fig. 1b, Fig. 4) [15]. In contrast, our model for *PIF/Pong/Harbinger* transposition is that they are mobilized by staggered cleavage of the TSDs, producing three bases of microhomology that facilitates NHEJ (Fig. 4). Based on this, we predicted that changing the TSDs in such a way as to disrupt the microhomology would affect the quality and efficiency of *mPing’s* excision site repair.

**Fig. 5:**
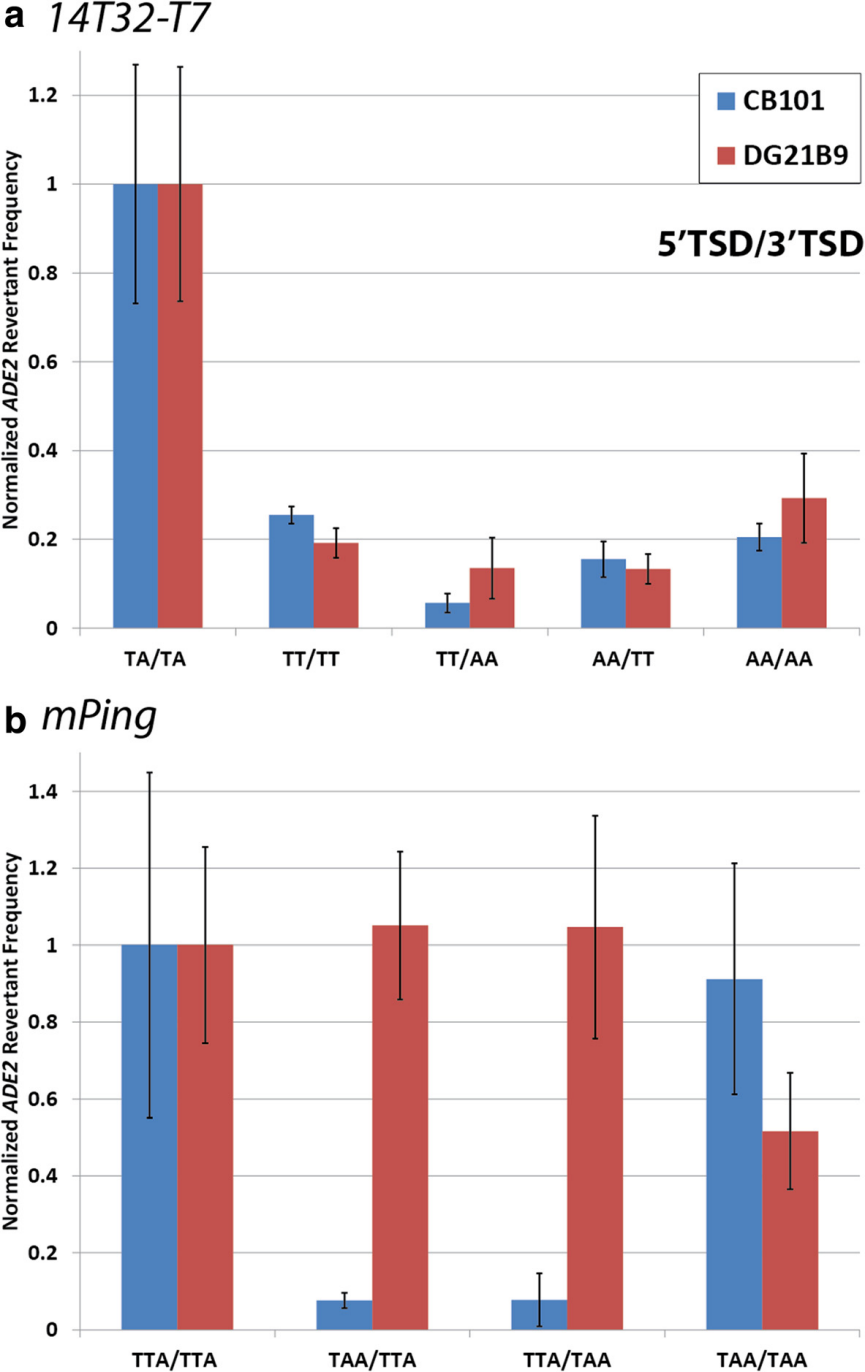
Transposition assays with non-matching TSDs. Normalized *ADE2* revertant frequencies for *14T32-T7* (**a**) and *mPing* (**b**) elements with altered TSDs. Blue bars indicate the rate in CB101 (capable of both NHEJ and HR), while red bars indicate the rate in DG21B9 (only capable of HR). Values were normalized to the wild-type TSD (left column). Error bars indicate the standard error

The fact that multiple bases are equally acceptable in the middle position of *mPing’s* TSDs allowed experiments to determine if homology between the two TSDs facilitates repair of *mPing* excision sites. Yeast transposition assays comparing *mPing* constructs with matching TSDs (TTA/TTA and TAA/TAA) and non-matching TSDs (TTA/TAA and TAA/TTA) were performed (Fig. 5, Additional file 3). As shown in CB101 (Fig. 5b) or JIM17 (Additional file 3) yeast strains, the *mPing* elements with non-matching TSDs showed significantly lower transposition than those with matching TSDs. Performing this assay in the DG21B9 strain, which is only capable of repair by HR did not show this effect, with all TSD combinations showing a similar number of *ADE2* revertant colonies (Fig. 5b). Together these results indicate that the reduction in *ADE2* revertant colonies for non-matching TSDs is caused by reduced or inaccurate NHEJ repair efficiency. For comparison, similar experiments using the *14T32-T7* element showed that non-matching TSDs produced a similar effect in both NHEJ competent (CB101) and NHEJ deficient (DG21B9) strains (Fig. 5a). This indicates that changing the TSDs of *14T32-T7* only affected its excision and not the repair of the excision site.

**Fig. 6:**
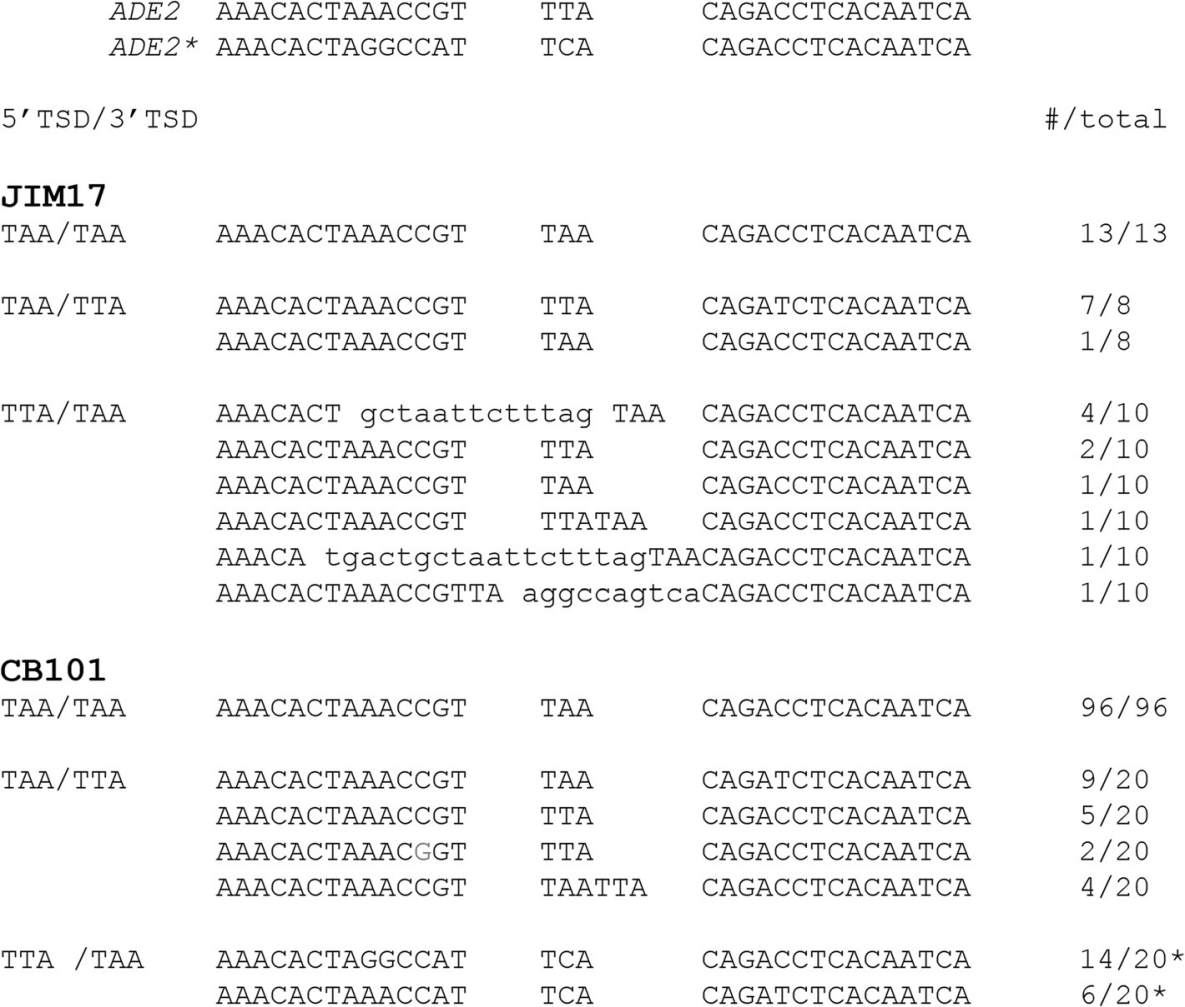
*mPing* excision sites for non-matching TIRs. Sequences identified at the *mPing* excision sites by restriction site analysis and sequencing in JIM17 (NHEJ only) and CB101 (HR and NHEJ). Lowercase letters indicate inserted sequences and a base change is in red. * indicates that the site was repaired by HR using the ADE2* template

Analysis of the excision sites from non-matching TSDs by restriction digest and sequencing was performed to determine how these sites were repaired. Figure 6 shows that in JIM17 and CB101 the excision site produced by an element with TAA/TTA TSDs was repaired fairly precisely with most excision sites only showing one of the TSDs. However, two of the excision sites retained both TSDs, consistent with staggered cleavage at the TSDs that was repaired by NHEJ without microhomology. In contrast, we found that repair of the excision site produced by an element with TTA/TAA TSDs was repaired less precisely in JIM17, or exclusively by HR repair using the ADE2* template in CB101 (Fig. 6). This result suggests that the staggered ends created by the TTA/TAA combination were not as easily joined by NHEJ pathway as the TAA/TTA combination. Since a 5′ overhang would create a different set of mismatched bases than a 3′overhang at the excision site (Table 1), we compared our results to the expected base pairing for each non-matching TSD. Based on this result, we propose that *mPing’s* TSDs cleavage produces a 5′ overhang (Fig. 4, Table 1). A three base 5′ overhang would result in the TAA/TTA TSDs forming a T:T (pyrimidine:pyrimidine) pairing at the middle base of the overhang, a more compatible pairing than the A:A (purine:purine) base paring created by the TTA/TAA TSDs.

**Table 1:** Base pairing that results after 5′ or 3′ staggered cleavage of the *mPing* TSDs

	Proposed middle base pairing	
*mPing* target site duplications	5′ overhang	3′ overhang
TTA/TTA	A:T	T:A
TTA/TAA	A:A	T:T
TAA/TTA	T:T	A:A
TAA/TAA	T:A	A:T

Based on this model, we should see that some TSD combinations are more detrimental to excision site repair than others. In fact, analysis of additional combinations of *mPing* TSDs (TCA and TGA) showed that non-matching TSDs, that according to our model would result in T:C (pyrimidine:pyrimidine) or A:G (purine:purine) mismatches, produced fewer *ADE2* revertants than TSD combinations that produce C:A (pyrimidine:purine) and T:G mismatches (pyrimidine:purine) (Additional file 4). Sequence analysis of the excision sites produced by selected TCA and TGA mismatched TSDs (Supplemental 4c) indicates that, for the most part, only one of the TSD sequences is left behind, as is expected of precise repair. However, about 14 % of the time both of the TSDs remained, leaving a footprint. This is in stark contrast to *mPing* elements with matching TSDs, which have never been observed to leave behind both TSDs upon excision (Fig. 2b) [20, 27].

It is not clear how common the excision site creation and repair mechanisms observed for *mPing* are present in other transposon superfamilies. Interestingly, alteration of the *P-element* TSDs from *Drosophila* showed a reduction in transposition activity [28]. Also, a recent study with the *Os3378* element (*Mutator* superfamily from rice) that also excises precisely, indicated that alteration of its TSDs reduces the rate of precise excision in yeast [29]. Analysis of these elements in the CB101 and DG21B9 yeast strains would be able to determine if this is due to disruption of excision or excision site repair.

### *mPing* TSDs do not influence target site insertion

Previous research has shown that *mPing* exhibits a strong preference for insertion into TAA or TTA sequences in the genome [20, 27, 30]. This is consistent with the findings of this study indicating that these sequences are required for efficient excision of the element. However, it was not known if the TSD sequences might play a role in the insertion preference of the element. To address this, 46 insertions of an *mPing* element with TCA/TCA TSDs were analyzed by sequencing transposon display PCR products [31]. We observed that 45 of the insertions were in TTA or TAA, and only one was in TCA. This is consistent with the results observed for the wild type *mPing* element [20], suggesting that the TSDs do not play a large role in target site selection.

## Conclusions

These results demonstrate a key difference in the transposition mechanisms used by the *Tourist*-like and *Stowaway*-like MITEs. While the excision sites of both *mPing* and *14T32* elements are primarily repaired by the NHEJ pathway in yeast, the *14T32* element appears to be more sensitive to alteration of NHEJ pathway genes. Our study suggests that the TSDs flanking both elements are required for their efficient excision. On the other hand, complementarity of the two TSDs was found only to be critical to the efficiency and precision of *mPing’s* excision site repair. Based on this finding, we conclude that the transposases that excise *mPing,* and presumably other *Tourist*-like MITEs, produce a staggered cut at the TSDs that provides microhomology that facilitates precise repair of the excision site.

## Methods

### Yeast strains and vectors

**Table:** 

**Strain name**	**Genotype**
JIM17	*MAT*a *ade2∆::hphMX4 his3∆1 leu2∆0 met15∆0 ura3∆0*
CB101	*MAT*a ade2∆::hphMX4 his3∆1 leu2∆0 met15∆0 ura3∆0 lys2∆::ADE2*
JIM16	*MAT*a *rad50∆::kanMX4 ade2∆::hphMX4 his3∆1 leu2∆0 met15∆0 ura3∆0*
JIM22	*MAT*a *mre11∆::kanMX4 ade2∆::hphMX4 his3∆1 leu2∆0 met15∆0 ura3∆0*
JIM21	*MAT*a *ku70∆::kanMX4 ade2∆::hphMX4 his3∆1 leu2∆0 met15∆0 ura3∆0*
DG21B9	*MAT*a ku70∆::kanMX4 ade2∆::hphMX4 his3∆1 leu2∆0 met15∆0 ura3∆0 lys2∆::ADE2*

*Saccharomyces cerevisiae* strains, BY4741 (JIM17) or Yeast Deletion Project strains [32, 33] in the BY4741 background (JIM16, JIM22, JIM21), were adapted for the study by deleting the *ADE2* gene using the *hphMX4 (pAG32)* cassette replacement technique [34] using the following primers: *ADE2hphMX* For-CAATCAAGAAAAACAAGAAAATCGGACAAAACAATCAAGTCCTTGACAGTCTTGACGTGC, *ADE2hphMX* Rev-ATAATTATTTGCTGTACAAGTATATCAATAAACTTATATACGCACTTAACTTCGCATCTG.

The partial *ADE2* template (ADE2*) was synthesized with the following sequence 5′-TTTGGCATACGATGGAAGAGGTAACTTCGTTGTAAAGAATAAGGAAATGATTCCGGAAGCTTTGGAAGTACTGAAGGATCGTCCTTTGTACGCCGAAAAATGGGCACCATTTACTAAAGAATTAGCAGTCATGATTGTGAGATCTGTGAATGGCCTAGTGTTTTCTTACCCAATTGTAGAGACTATCCACAAGGACAATATTTGTGACTTATGTTATGCGCCTGCTAGAGTTCCGGACTCCGTTCAACTTAAGGCGAAGTTGTTGGCAGAAAATGCAATCAAATCTTTT-3′ and cloned between the *Bgl*II and *Hind*III sites of the pIS 385 disintegrator plasmid [35]. To make the CB101 and DG21B9 yeast strains, this plasmid was then linearized with *Nru*I (New England Biolabs, Massachusetts, USA) and transformed into the *LYS2* locus of JIM17 and JIM21, respectively. Selection and screening were performed as described [35] to remove the *URA3* selectable marker and identify transformants that maintained the genomic copy of the ADE2* template.

The pAG413 *Pong* ORF1, pAG415 *Pong* transposase L418A, L420A and pWL89A *mPing* plasmids were described previously [20]. The pAG415 *Osmar14* transposase was made by PCR amplification of the open reading frame from a previously described *Osmar14* transposase plasmid [21] with the following primers *Osmar 14* For – GGGGACAAGTTTGTACAAAAAAGCAGGCTTCATGCAAGAGTACGGCGTGTATGC, *Osmar 14* Rev- GGGGACCACTTTGTACAAGAAAGCTGGGTCTTAAACTGCACTTGGTTGGCTAATGCT. The PCR product was inserted into the Gateway® pDONR™/Zeo vector using a BP clonase reaction (Life Technologies, Carlsbad, CA), then transferred into pAG415 GAL ccdb using an LR clonase Reaction (Life Technologies, Carlsbad, CA). The reporter plasmids pwL89A *14T32* and *14T32-T7* were described previously [14, 21]. TSD mutations were made to the MITEs *mPing* and *Osmar 14T32-T7* by PCR amplification using primers altered at the TSD (underlined positions indicate TSD), for example:

*mPing* TGA For – AGTCTCTACAATTGGGTAAGAAAACACTAAACCGT**TGA***GGCCAGTCACAATGGGGGTTTC*

*mPing* TGA Rev – ACTAAAGAATTAGCAGTCATGATTGTGAGGTCTG**TCA***GGCCAGTCACAATGGCTAGTGTC*

*14T32* AT For –CTAAAGAATTAGCAGTCATGATTGTGAGGTCTGTT**AT***CTCCCTCCGTCCCAGAAAGAAGG*, and

*14T32* AT Rev – GTCTCTACAATTGGGTAAGAAAACACTAAACCGTT**AT***CTCCCTCCGTCCCAGAAAGAAGC*

The resulting PCR products were purified using a clean and concentrate kit (Zymo Research, Irvine, CA) and then transformed together with *Hpa*I digested pWL89A using the LiAc method [36]. Mutations were verified by sequencing PCR products or purified plasmids with following primers that flank the *ADE2 Hpa*I site: *ADE2*-CF-GGGTTTTCCATTCGTCTTGAAGTCGAGGAC and *ADE2*-CR-CATTTCCACACCAAATATACCACAACCGGGA.

### Yeast transposition assay

Transposition assays were performed using two techniques depending on the relative transposition rates. For low activity combinations (i.e. Figs. 1, 3 and 4b, and Additional files 3 and 4) transformed yeast were grown in 5 ml of selective media (2 % dextrose) at 30 °C for 48 h, centrifuged to concentrate the culture, plated on selective 2 % galactose plates (150 mm) lacking adenine, and incubated at 30 °C for 15 days as described [20]. For experiments with higher rates of transposition (i.e. Fig. 2, 5a and Additional file 1), a 3 ml liquid (2 % dextrose) culture was grown for 24 h at 30 °C and 100 μl was plated on selective 2 % galactose plates (100 mm) and incubated at 30 °C for 10 days. A time course of this procedure showed that the number of *ADE2* revertant colonies had a linear rate of appearance (Additional file 1). Dilution series of the liquid cultures plated on complete YPD media were used to determine the total number of cells plated. Transposition rate was calculated by dividing the number of *ADE2* revertant colonies by the total number of yeast plated.

### Excision site analysis

*ADE2* revertant colonies were suspended in 20 μl of 1 unit/μl Zymolyase (Zymo Research, Irvine, CA) and incubated for 15 min at 37 °C to lyse the yeast cells. PCR amplification of the excision site was performed using the ADE2-CF and ADE2-CR primers in a 20 μl reaction with 2 μl of lysed yeast as the template. PCR products were diluted and digested with *HpaI* or *HaeIII* (New England Biolabs, Massachusetts, USA) and then analyzed by agarose gel electrophoresis. PCR products were treated with ExoSAP-IT (USB Corporation, Ohio, USA) per instruction of the manufacturer prior to sequencing.

### Insertion site analysis

Transposon display analysis of *mPing* insertion sites were performed as described previously [20, 30, 31]. Individual bands were sequenced after cutting them from the gel and performing PCR amplification with the transposon display primers.

## Additional files

Additional file 1:
**Time course of a yeast transposition assay.** *ADE2* revertant frequencies for *mPing* in the JIM17 strain of yeast. (PDF 305 kb) Additional file 2:
**Additional altered matching TSDs.** Additional examples of the of *ADE2* revertant frequency for *mPing* elements with alternative but matching TSDs and their associated excision site sequences. (PDF 360 kb) Additional file 3:
**Non-matching** ***mPing*** **TIRs in JIM17.** Chart comparing the frequency of *ADE2* revertant colonies produced for *mPing* elements with matching (i.e. TAA/TAA) and non-matching TSD sequences (i.e. TAA/TTA) in the JIM17 strain. (PDF 403 kb) Additional file 4:
**Additional non-matching** ***mPing*** **TIRs.** Charts comparing the frequency of *ADE2* revertant colonies produced for *mPing* elements with additional combinations of matching (i.e. TCA/TCA) and non-matching TSD sequences (i.e. TAA/TCA) in the JIM17 strain. (PDF 593 kb)

## Abbreviations

TE: Transposable element
MITE: Miniature inverted repeat transposable element
TSD: Target site duplication
TIR: Terminal inverted repeat
NHEJ: Non-homologous end joining
HR: Homologous recombination
