# Supplementary material for: Precise repair of mPing excision sites is facilitated by target site duplication derived microhomology
Source: Mob DNA. 2015 Sep 7;6:15. doi: 10.1186/s13100-015-0046-4 (PMC4561436; doi:10.1186/s13100-015-0046-4)

Precise repair of *mPing* excision sites is facilitated by target site duplication derived microhomology

David M. Gilbert, M. Catherine Bridges, Ashley E. Strother, Courtney E. Burckhalter, James M. Burnette III, and C. Nathan Hancock

Additional file 1

Time course of a yeast transposition assay

ADE2 revertant frequencies for *mPing* in the JIM17 strain of yeast. Error bars represent the standard error for six replicates.

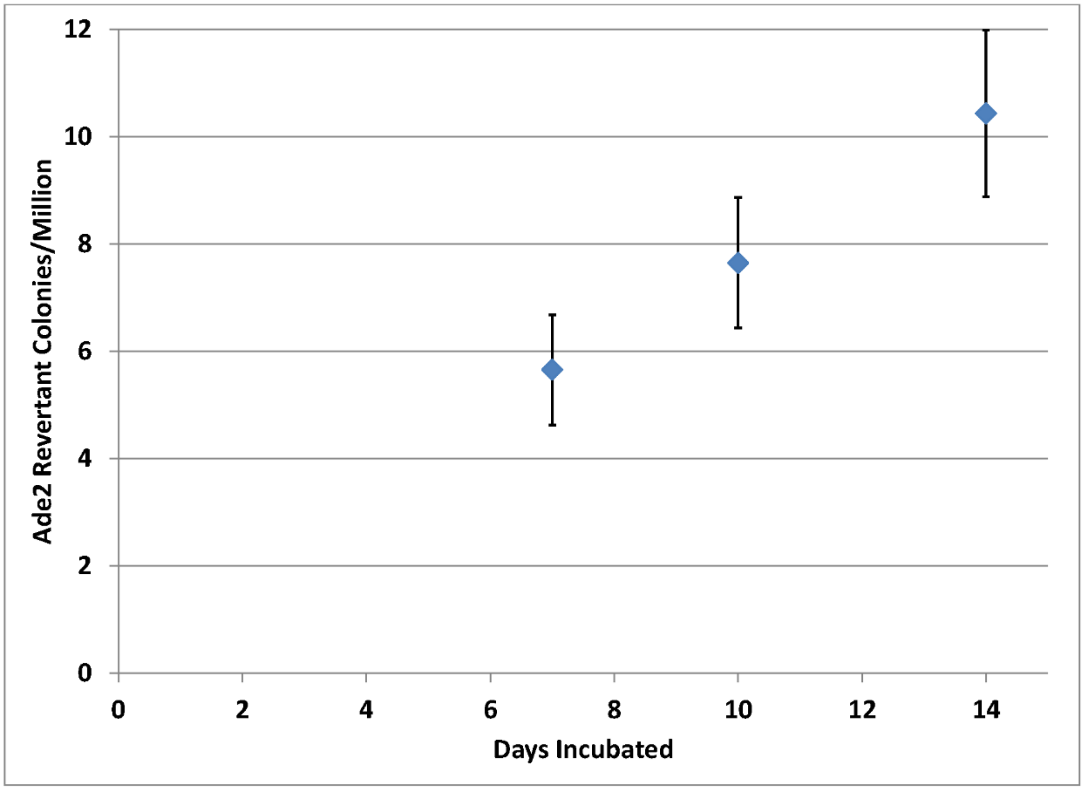

Supplement: Additional file 1: — Time course of a yeast transposition assay. ADE2 revertant frequencies for mPing in the JIM17 strain of yeast. (PDF 305 kb) [file 13100_2015_46_MOESM1_ESM.pdf]
