# Supplementary material for: Precise repair of mPing excision sites is facilitated by target site duplication derived microhomology
Source: Mob DNA. 2015 Sep 7;6:15. doi: 10.1186/s13100-015-0046-4 (PMC4561436; doi:10.1186/s13100-015-0046-4)
Supplement: Additional file 2: — Additional altered matching TSDs. Additional examples of the of ADE2 revertant frequency for mPing elements with alternative but matching TSDs and their associated excision site sequences. (PDF 360 kb) [file 13100_2015_46_MOESM2_ESM.pdf]

Precise repair of *mPing* excision sites is facilitated by target site duplication derived microhomology

David M. Gilbert, M. Catherine Bridges, Ashley E. Strother, Courtney E. Burckhalter, James M. Burnette III, and C. Nathan Hancock

**Additional file 2**

**Additional altered matching TSDs**

Chart showing the frequency of *ADE2* revertant colonies produced for *mPing* elements with alternative but matching TSDs (a). TSDs are represented as 5'TSD/3'TSD. Assays were performed in JIM17. Results are normalized to TAA/TAA and the error bars represent the standard error of six replicates. Sequences of the excision sites recovered from *ADE2* revertant colonies produced by *mPing* elements with altered TSDs (b). Inserted bases are shown as lowercase letters.

a)

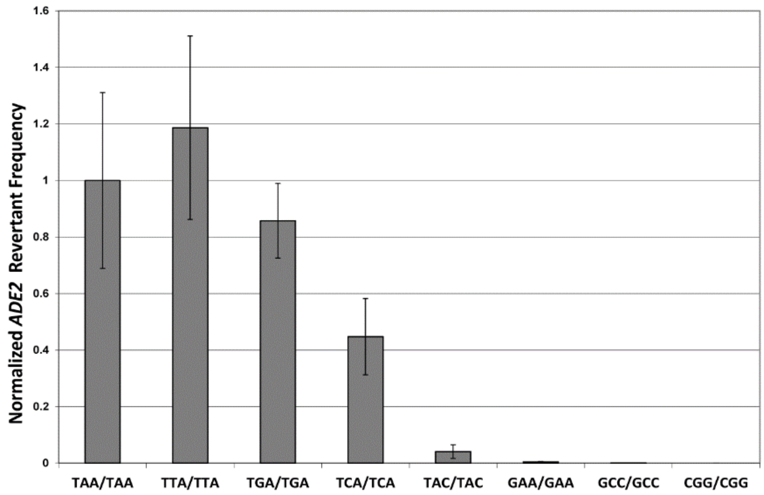


b) ***mPing* Excision Sites**

```
ADE2
   CACTAAACCGT TAA    CAGACCTCACAA

5'TSD/3'TSD                        #/Total
TCA/TCA
   CACTAAACCGT TCA    CAGACCTCACAA 25/25

TGA/TGA
   CACTAAACCGT TGA    CAGACCTCACAA  23/24
   CAATAAACCGT cGA    CAGACCTCACAA  1/24

TAC/TAC
   CACTAAACCGT TAC    CAGACCTCACAA  6/16
   CACTAAACCGT tataaa CAGACCTCACAA  5/16
   CACTAAAC gggtaacaaaacc    CACAA  2/16
   CACTAAACCGT tataaa CAGACCTCACAA  1/16
   CACTAAACCGg TAC    CAGACCTCACAA  1/16
   CACTAAACCGT TAC ggccagtccaa   A  1/16
```
