# Supplementary material for: Precise repair of mPing excision sites is facilitated by target site duplication derived microhomology
Source: Mob DNA. 2015 Sep 7;6:15. doi: 10.1186/s13100-015-0046-4 (PMC4561436; doi:10.1186/s13100-015-0046-4)

Precise repair of *mPing* excision sites is facilitated by target site duplication derived microhomology

David M. Gilbert, M. Catherine Bridges, Ashley E. Strother, Courtney E. Burckhalter, James M. Burnette III, and C. Nathan Hancock

**Additional file 3**

**Non-matching *mPing* TIRs in JIM17**

Chart comparing the frequency of *ADE2* revertant colonies produced for *mPing* elements with matching (i.e. TAA/TAA) and non-matching TSD sequences (i.e. TAA/TTA) in the JIM17 strain. The TSD sequence is represented as 5'TSD/3'TSD and the proposed middle base pairing associated with each combination is shown in parenthesis.  Results were normalized to TAA/TAA and error bars represent standard error of 6 replicates.

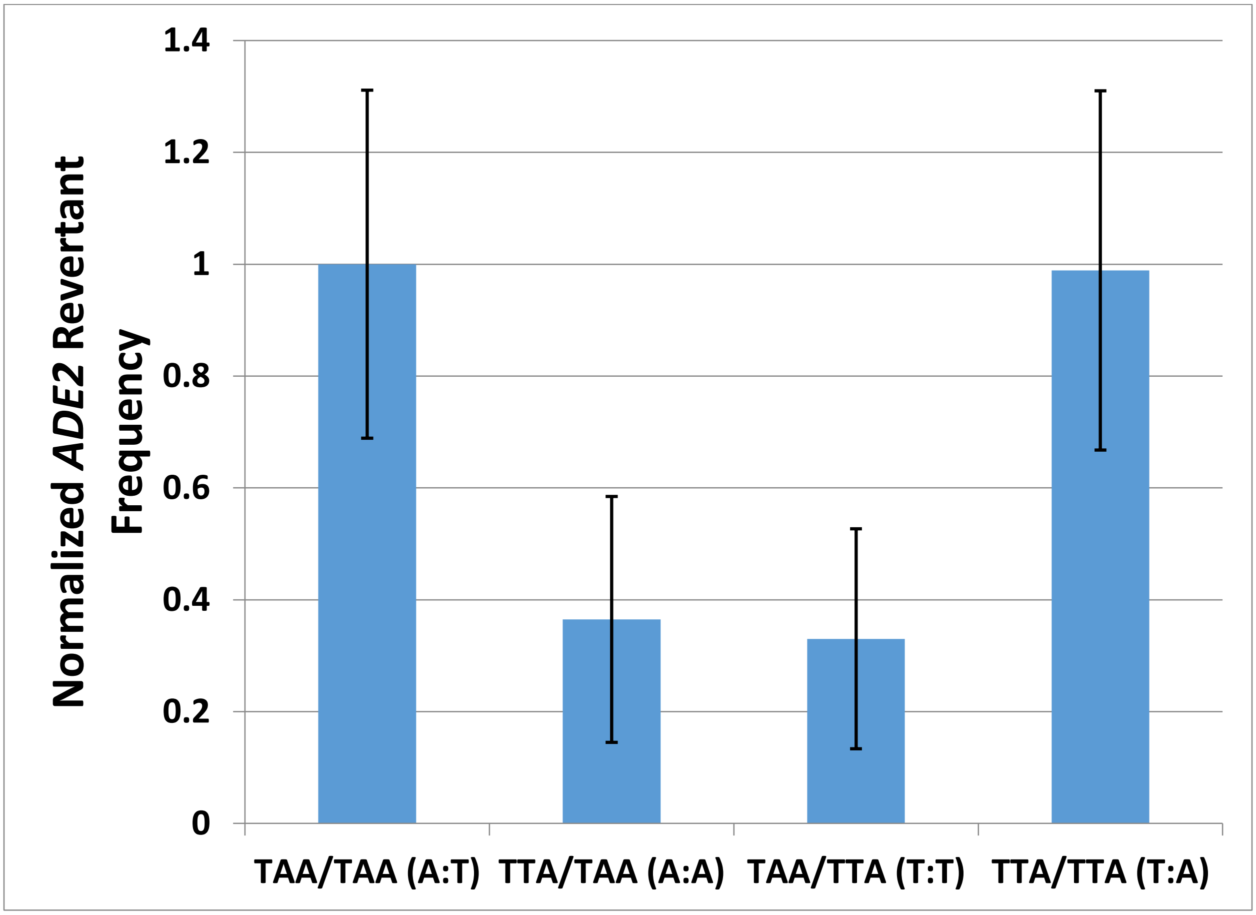

Supplement: Additional file 3: — Non-matching mPing TIRs in JIM17. Chart comparing the frequency of ADE2 revertant colonies produced for mPing elements with matching (i.e. TAA/TAA) and non-matching TSD sequences (i.e. TAA/TTA) in the JIM17 strain. (PDF 403 kb) [file 13100_2015_46_MOESM3_ESM.pdf]
