# Supplementary material for: Precise repair of mPing excision sites is facilitated by target site duplication derived microhomology
Source: Mob DNA. 2015 Sep 7;6:15. doi: 10.1186/s13100-015-0046-4 (PMC4561436; doi:10.1186/s13100-015-0046-4)
Supplement: Additional file 4: — Additional non-matching mPing TIRs. Charts comparing the frequency of ADE2 revertant colonies produced for mPing elements with additional combinations of matching (i.e. TCA/TCA) and non-matching TSD sequences (i.e. TAA/TCA) in the JIM17 strain. (PDF 593 kb) [file 13100_2015_46_MOESM4_ESM.pdf]

Precise repair of *mPing* excision sites is facilitated by target site duplication derived microhomology

David M. Gilbert, M. Catherine Bridges, Ashley E. Strother, Courtney E. Burckhalter, James M. Burnette III, and C. Nathan Hancock

Additional file 4

Additional non-matching *mPing* TIRs

Charts comparing the frequency of *ADE2* revertant colonies produced for *mPing* elements with matching (i.e. TCA/TCA) and non-matching TSD sequences (i.e. TAA/TCA) in the JIM17 strain. Combinations using TCA TSDs (a) and combinations using TGA TSDs (b) are shown. The TSD sequence is represented as 5'TSD/3'TSD and the proposed middle base pairing that results from 5' overhangs associated with each combination is shown in parenthesis. Results were normalized to matching TSDs (i.e. TCA/TCA) and error bars represent standard error. Sequences of the excision sites recovered from selected *ADE2* revertant colonies produced by *mPing* element with non-homologous TSDs (c). * indicates a recovered excision site sequence that includes a frame shift.

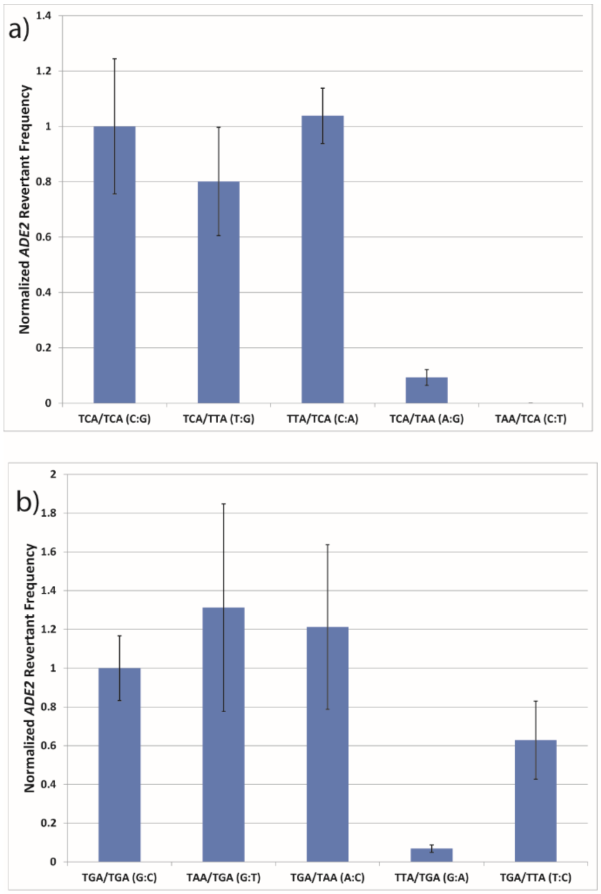

c) ***mPing* Excision Sites**

```
ADE2
   CACTAAACCGT TAA    CAGACCTCACAA

5'TSD/3'TSD                        #/Total
TAA/TGA
   CACTAAACCGT TAA    CAGACCTCACAA 5/10
   CACTAAACCGT TGA    CAGACCTCACAA 2/10
   CACTAAACCGT TAATGA CAGACCTCACAA 2/10
   CACTAAACCGT A TGA  CAGACCTCACAA 1/10*
TGA/TAA
   CACTAAACCGT TAA    CAGACCTCACAA 3/6
   CACTAAACCGT TGA    CAGACCTCACAA 3/6
TTA/TCA
   CACTAAACCGT TCA    CAGACCTCACAA 22/27
   CACTAAACCGT TTA    CAGACCTCACAA 2/27
   CACTAAACCGT TTATCA CAGACCTCACAA 2/27
   CAATAAACCGT TCA    CAGACCTCACAA 1/27
TCA/TTA
   CACTAAACCGT TTA    CAGACCTCACAA 15/22
   CACTAAACCGT TCATTA CAGACCTCACAA 5/22
   CACTAAACCGT TCA    CAGACCTCACAA 2/22
```
